# Navigating the coronavirus pandemic 2 years on: Experiences of people with dementia from the British IDEAL cohort

**DOI:** 10.1177/14713012231158215

**Published:** 2023-02-24

**Authors:** Eleanor Dawson, Rachel Collins, Claire Pentecost, Sally Stapley, Catherine Quinn, Catherine Charlwood, Christina Victor, Linda Clare

**Affiliations:** University of Exeter Medical School, 3286 University of Exeter, Exeter, UK; Centre for Applied Dementia Studies, 1905 University of Bradford, Bradford, UK; Wolfson Centre for Applied Health Research, Bradford, UK; University of Exeter Medical School, 3286 University of Exeter, Exeter, UK; College of Health, Medicine and Life Sciences, 3890 Brunel University London, UK; University of Exeter Medical School, 3286 University of Exeter, Exeter, UK; NIHR Applied Research Collaboration South-West Peninsula, Exeter, UK

**Keywords:** COVID, Alzheimer’s, qualitative, coping, isolation

## Abstract

**Background and objectives:**

People with dementia have been affected in unique ways during the COVID-19 pandemic. It is not known whether the impact of the pandemic has changed with time or with the changes in social restrictions. This study explored how experiences of coping with the effects of the pandemic in the UK changed over time.

**Research design and methods:**

We conducted semi-structured interviews with people with dementia living in the community in England and Wales who had taken part in a qualitative interview at an earlier stage of the pandemic. We applied framework analysis to identify themes and compared these with interviewees’ previous accounts.

**Findings:**

Nine people aged between 51 and 89 years were interviewed; four were female and five had early onset dementia. We identified three themes: 1. Navigating a changing world: Living with coronavirus; 2. A ‘downward spiral’: Managing advancing dementia; and 3. Availability, accessibility, and suitability of support. Findings reflect participants’ ongoing caution about re-emerging from social restrictions to resume valued activities, and how this led to coping behaviours to minimise the impact on wellbeing in the absence of formal support and services.

**Discussion and implications:**

Despite easing of restrictions across the UK, the negative impact of the coronavirus pandemic on people with dementia continues. Whilst individuals and services have adapted to some of the challenges, there is now an opportunity to rebuild support networks and services to ensure people with dementia are suitably advised, supported and socially engaged to allow them to live as well as possible.

## Background and objectives

Globally, more than 55 million people are living with dementia (World Health Organisation, 2021). In order to ‘live well’, many individuals employ coping strategies, pursue meaningful activities, and develop support networks, for example by joining dementia support groups (Weetch et al., 2021). For those living in the community, these adaptations are fundamental in providing a sense of purpose and facilitating continued independence and identity (Alzheimer’s Society, 2022). The outbreak of the SARS-CoV-2 coronavirus in Wuhan, China, in December 2019 and subsequent declaration of a pandemic in March 2020 (World Health Organisation, 2020) led many countries to implement stringent public health measures to limit viral transmission, such as the wearing of face masks and social isolation. For people with dementia, restrictions introduced in Britain resulted in closures of vital support groups, limited opportunities for social contact, and reduced access to healthcare services (Bannon et al., 2021; Clare et al., 2022; Giebel et al., 2021; Rising et al., 2022; Tuijt et al., 2021). Moreover, the pandemic-enforced shift towards telemedicine in Britain served to amplify existing inequalities in service provision, in addition to suitability and access concerns (O'Rourke et al., 2021; Tuijt et al., 2021).

A systematic review of cross-sectional studies conducted early in the pandemic found these social restrictions adversely affected people living with dementia, with declines observed in physical health, behavioural and psychological symptoms, independence, and functional ability (Suárez-González et al., 2021). Of particular concern were perceived losses in cognitive and communicative abilities resulting from fewer opportunities to interact socially and practise skills; these fears were evident in both quantitative and qualitative studies (Borelli et al., 2021; Boutoleau-Bretonnière et al., 2020; Brown et al., 2020; Canevelli et al., 2020; O'Rourke et al., 2021; Talbot & Briggs, 2021). Social restrictions were also associated with increased isolation and loneliness (Banerjee & Rai, 2020; Hanna et al., 2021), decreased physical activity (Di Lorito et al., 2021), and depression (Banerjee & Rai, 2020; Rising et al., 2022). Taken together, these changes have the potential to negatively influence the ability to ‘live well’ with dementia (Clare et al., 2022).

However, while these adverse impacts are certainly cause for concern, comparative and matched longitudinal studies suggested a limited impact of the pandemic after one year of restrictions (Clare et al., 2022; Sabatini et al., 2022). Self-report and informant data collected from the IDEAL cohort before and during the pandemic indicated little negative impact on the physical health, mood, social connections and relationships of people with dementia (Clare et al., 2022). Indeed, contrary to earlier cross-sectional evidence, prevalence of depression and anxiety had decreased compared to pre-pandemic levels, with reports of increasing optimism and satisfaction with support from family members. Authors attribute these findings to the resilience of people with dementia in learning to adapt to an evolving context of coronavirus, for example by implementing new daily routines, spending time in nature, and communicating with friends, family, and health and social care providers through remote methods (Clare et al., 2022; Sabatini et al., 2022). Several qualitative studies have reported the use of such strategies to aid coping (O'Rourke et al., 2021; Pentecost et al., 2022; Portacolone et al., 2021; Rising et al., 2022; Stapley et al., 2022). Notably, however, these qualitative studies suggest the impact of the pandemic on people with dementia is far more variable and nuanced than much of the quantitative evidence suggests. Varying access to support, social interactions, and personal capacities in managing everyday life with dementia and coronavirus have understandably affected individuals differently (Cousins et al., 2021). Therefore, while quantitative cross-sectional and longitudinal studies are useful in presenting experiences from larger cohorts, qualitative studies can provide additional depth in understanding beliefs, attitudes, and experiences during this turbulent period. However, most of the available qualitative studies were conducted in the early stages of the pandemic and there is a need to better understand how early impacts on physical, cognitive, and psychosocial wellbeing have evolved over time.

The present study therefore adopted a qualitative approach to explore how people with dementia were coping after 22 months of social restrictions in England and Wales. This is the first qualitative study to follow the experiences of people with dementia during changing social restrictions over a significant time-period. Specifically, we sought to understand whether previous coping strategies had been adapted as restrictions eased; whether attitudes towards the pandemic, restrictions, and risk had changed; and whether there was adequate health and social support in place to navigate the complex and evolving reality of the pandemic. Uniquely, we can offer an in-depth contextual perspective by re-contacting individuals interviewed during the first year of restrictions from May to July 2020, November to December 2020 and January to April 2021 and interviewing them again during the emergence of the new Omicron variant in Britain from December 2021 to January 2022. With this approach, we can offer novel insight into the ongoing impact and emerging needs of people with dementia during the pandemic that could help prepare for similar social health measures in the future.

## Research Design and Methods

### Design

This qualitative study forms part of the INCLUDE project (Identifying and mitigating the individual and dyadic impact of COVID-19 and life under physical distancing on people with dementia and caregivers; Clare et al., 2022; Quinn et al., 2022), a component of the British IDEAL cohort study (Clare et al., 2014; Silarova et al., 2018) intended to explore the experiences of people with dementia and their caregivers in England and Wales during the coronavirus pandemic using both quantitative and qualitative methods. INCLUDE built upon in-depth interviews conducted between May and July 2020 as part of the IDEAL COVID-19 Dementia Initiative (IDEAL-CDI)(O'Rourke et al., 2021) and was developed in collaboration with the IDEAL public and patient involvement group, ALWAYs. For a description of the IDEAL, IDEAL CDI and INCLUDE studies see Table 1. INCLUDE participants completed structured telephone or online interviews between September 2020 and April 2021 (Clare et al., 2022; Quinn et al., 2022), and a subset of participants additionally engaged in qualitative semi-structured interviews conducted either before (November to December 2020) (Pentecost et al., 2022) or after (January to April 2021) (Stapley et al., 2022) the start of the vaccination programme in England and Wales.

**Table 1.** IDEAL, IDEAL-2 and INCLUDE cohort studies.

Study	Acronym definition	Description
IDEAL (2014-2019)	Improving the experience of dementia and enhancing active life	A large cohort study with a quantitative arm focusing on facilitators of, and challenges to living well with dementia for people with dementia and their families and how this changes over time as dementia progresses. Survey interview data was collected when participants joined the study, and again one and 2 years later
IDEAL-2 (2018-2022)	Improving the experience of dementia and enhancing active life - 2	A continuation of the IDEAL cohort study, following IDEAL participants for three additional time points over a 3-year period
IDEAL-CDI (2020)	IDEAL COVID-19 dementia Initiative	A COVID-19 qualitative sub-component of the IDEAL programme, established in April 2020
INCLUDE (2020-2022)	Identifying and mitigating the individual and dyadic impact of COVID-19 and life under physical distancing on people with dementia and carers	A sub-component of IDEAL. A survey with a small qualitative arm designed to examine the impact of the COVID-19 pandemic and resulting restrictions on IDEAL participants. Structured interview data were collected once during the second wave of the pandemic Sep 2020-April 2021. Semi-structured qualitative data were collected at 3 time-points: 1) before and 2) after commencement of the UK vaccination program in late 2020/early 2021 and 3) (the current study) during restrictions to protect from the emerging new Omicron variant in late 2021-early 2022

**Figure 1.**
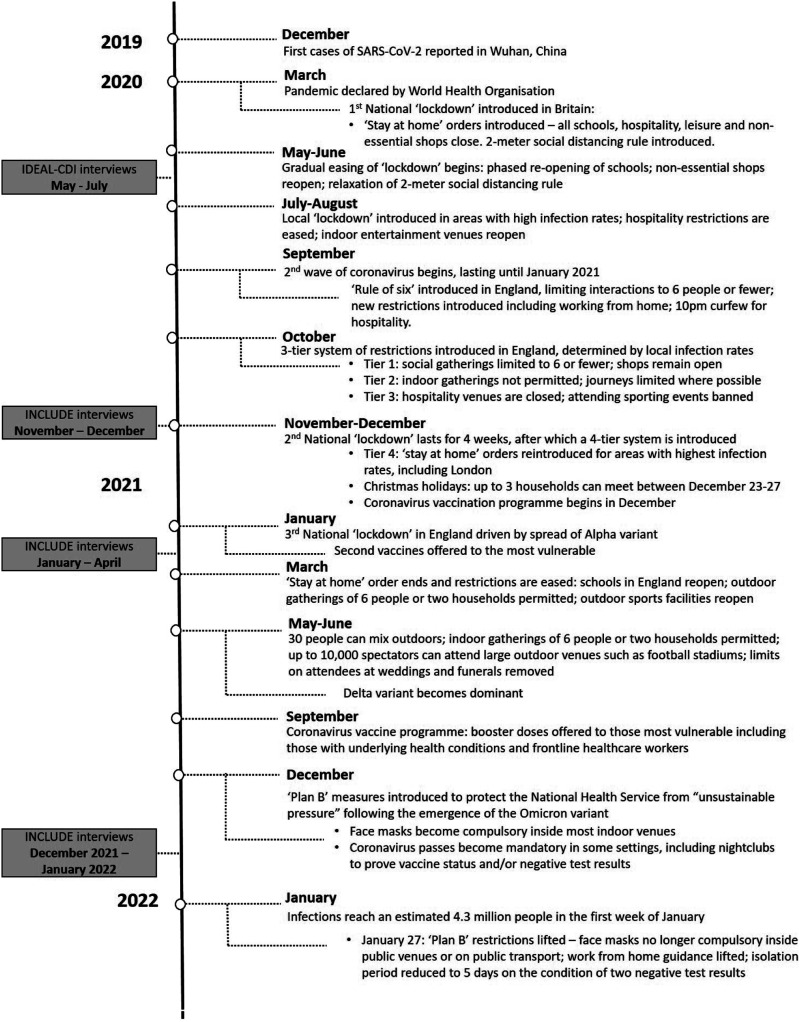
Timeline of public health restrictions in England and Wales between March 2020 and January 2022 and stages of data collection.

Here, we report findings from a further round of qualitative semi-structured interviews with people with dementia conducted between December 13, 2021, and January 25, 2022, after the programme to deliver second vaccinations was completed and during the emergence of the new Omicron variant in England and Wales. On December 8, 2021, measures were introduced in England to limit transmission of the new Omicron variant and to protect the National Health Service (NHS) from becoming overwhelmed. These measures included wearing face masks inside public venues and working from home. Coronavirus infections reached 4.3 million cases in the week ending January 9, 2022 (Office for National Statistics, 2022). See Figure 1 for a timeline of the pandemic restrictions in relation to timing of study interviews. Caregivers’ experiences were explored in a parallel study reported separately.

INCLUDE was approved by Wales Research Ethics Committee 5 as an amendment to the IDEAL ethical approvals for England and Wales (18/WS/0111 AM12 and 18/WA/0111/AM14). IDEAL is registered with the UK Clinical Research Network (UKCRN), numbers 16593 and 37955.

### Participants and Procedures

**Figure 2.**
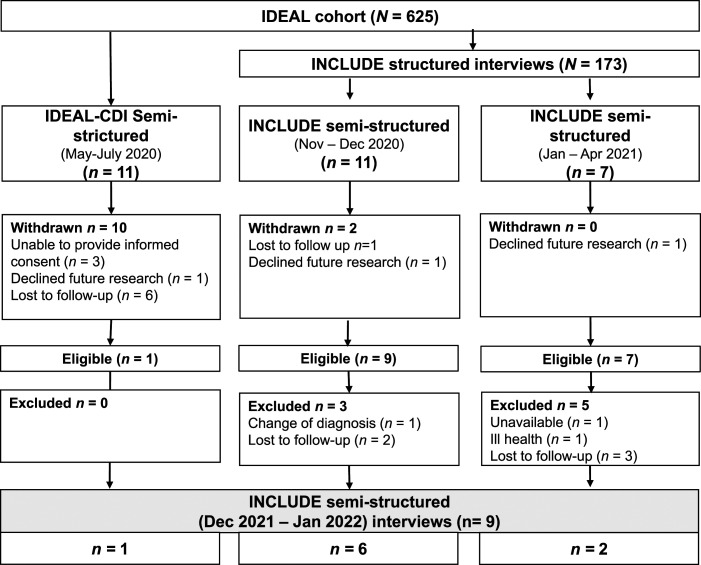
Numbers recruited from each of the three timepoints during the pandemic: Reasons for withdrawal prior to checking eligibility and further dropout prior to interviews.

We identified 29 people with dementia who had previously taken part in semi-structured interviews for the IDEAL-CDI or INCLUDE studies (see Figure 2 showing the numbers recruited from each of the previous three timepoints during the pandemic). Of these participants, 17 were eligible and had indicated a willingness to participate in future research and were contacted by telephone or email with information on the study. Inclusion was determined by availability and ability to provide verbal informed consent.

Interviews were conducted by either telephone or Zoom, according to participant preference. Informed consent was audio-recorded and a copy of the consent form was emailed or posted at the participants’ request. Interviews lasted between 23 and 45 min, and were conducted by ED, a trained graduate researcher experienced in conducting interviews with people with dementia. A topic guide, adapted from previous INCLUDE interviews, was used to explore ongoing experiences of the pandemic and how people felt about the emerging Omicron variant (see Supplementary Materials). Questions focused on changes in coping, routines, and social contact, access to support and information, and suggestions on how participants could be best supported by healthcare professionals. Personalised prompts were used to follow up on earlier experiences. These prompts were based on notes ED had made before starting the interviews, having read each participant’s IDEAL-CDI or INCLUDE transcript to aid familiarisation and personalise interview questions. All interviews were audio-recorded and professionally transcribed verbatim.

### Analysis

Framework analysis was used to guide thematic analysis of the transcripts (Gale et al., 2013) following the five stages of familiarisation, identification of a thematic framework, indexing, charting and mapping and interpretation. This was the same approach taken when analysing the earlier IDEAL-CDI and INCLUDE studies. Analysis was conducted by a core team consisting of ED, RC, CP and SS, all of whom had prior involvement in analysing data from IDEAL-CDI and/or INCLUDE and were therefore familiar with the participants and the topics derived from previous interviews.

Familiarisation involved the core analysis team listening to audio-recordings and reading and annotating transcripts. Transcripts and field notes made immediately after each interview were read alongside each participant’s IDEAL-CDI or INCLUDE interview and similarities and changes to previous experiences were noted.

The coding frameworks, themes and subthemes from previous analyses of INCLUDE and IDEAL-CDI qualitative data were used to form an initial deductive framework that was discussed and agreed with the wider team. This framework was applied to four randomly-selected transcripts and coded by ED using NVivo 12 (QSR International, 2020) and new inductive codes were added. ED checked both within and between cases of the four transcripts to monitor consistency of coding, iteratively refining and developing codes as required. ED and RC discussed and agreed the framework and code descriptors then ED continued to apply the devised framework to the remaining transcripts (indexing) while checking within and between all nine interviews. To ensure methodological rigour, CP reviewed the framework and independently coded four randomly selected transcripts to confirm its consistent application.

Charts of the coded text were created to scrutinise the data to identify themes and map any patterns in responses over time. Emerging themes and subthemes across timepoints were discussed with the whole team continually and agreed. Finally, key data were identified that informed recommendations to better support people with dementia in the future.

## Findings

Nine participants aged between 51 and 89 were interviewed, five of whom had young-onset dementia (see Table 2). Four were female, five lived alone and five were university educated or had professional qualifications. Alzheimer’s disease was the most common diagnosis (67%); two participants had frontotemporal dementia and one had mixed dementia. Time since diagnosis ranged from 3 to 8 years. A summary of participant characteristics can be found in Table 2.

**Table 2.** Participants’ clinical and sociodemographic characteristics.

ID	Age	Gender	Ethnicity	Dementia			Marital status	Living situation	Location (country)	Education
				Diagnosis	YOD^a^	Years since diagnosis				
P01	89	Male	White British	AD		7	Married	Alone	Suburban (E)	School leaving certificate at age 16
P02	66	Male	White British	Mixed (AD/VaD)	Yes	6	Divorced	Alone; Assisted living	Rural (E)	University degree
P03	51	Female	White British	PCA	Yes	6	Single	Alone	Suburban (E)	University degree
P04	76	Male	White other	AD		6	Divorced	Partner	Suburban (E)	University degree
P05	63	Female	White British	FTD^b^	Yes	8	Married	Spouse	Rural (E)	Vocational qualification
P06	68	Male	White British	AD	Yes	7	Married	Spouse	Rural (E)	Apprenticeship
P07	85	Female	White British	AD		6	Divorced	Alone	Rural (W)	University degree
P08	64	Female	White British	AD	Yes	7	Divorced	Alone	Rural (W)	Professional qualification
P09	71	Male	White British	FTD		3	Married	Spouse	Suburban (E)	School leaving certificate at age 16

AD: Alzheimer’s disease; VaD: vascular dementia; PCA: posterior cortical atrophy; FTD: frontotemporal dementia; E: England; W: Wales.

All participants were retired, except P04 who continues to work one morning per week.

^a^Young-onset dementia (YOD) refers to a diagnosis made before the age of 65 (Dementia UK, 2022).

^b^Behavioural variant.

Three key themes were derived from the analysis, reflecting the changing experiences of people living with dementia nearly 2 years into the pandemic: 1. Navigating a changing world: Living with coronavirus, 2. A ‘downward spiral’: Managing advancing dementia, and 3. Availability, accessibility and suitability of support. How participants’ experiences of the pandemic changed over time are outlined in Table 3. Further illustrative quotes are provided in Table 4.

**Table 3.** Comparison of participants’ experiences across the pandemic.

Participant ID	Key experiences from previous interview	Key experiences from current interview
	IDEAL-CDI (May-July 2020)	(Dec 2021 – Jan 2022)
	P01	• Acceptance of situation; comfort through religion • Lives with his wife who also has Alzheimer’s disease • Remote support from family • Welfare calls from a charity	• Increasingly isolated and lonely • Limited stimulation, apathetic; routine based solely on visiting and calling wife who now lives in a care home • Remote support from family and friends; practical help from his cleaner • Mobility severely impeded • Welfare calls from a charity, social services, and memory clinic
	Pre-Vaccine Rollout (Nov-Dec 2020)	(Dec 2021 – Jan 2022)
	P02	• In-person dementia groups are closed • Fear of decline in communication skills • Increased anxiety going out • Use of remote communication (social and dementia advocacy groups) • Helping others • Lacked reassurance from healthcare services and advice on effective coping strategies and staying safe	• In-person dementia groups are closed • Decline in communication skills; practicing in-person where possible • Anxiety remains high; avoiding others and restricting public transport use • Keeping busy and helping others • Emerging need for emotional support • Difficulties with healthcare provisions – delays, lack of adequate dementia training
P03	• Anxiety linked to dementia and lenient coronavirus restrictions • Developing skills and coping strategies at home • Weekly contact with a healthcare professional offering practical and emotional support • Use of remote communication (social and dementia advocacy groups)	• Increasing anxiety as dementia progresses and coronavirus restrictions have eased; avoiding others and restricting public transport use • Learning new skills to limit dementia decline • Dementia groups using blended approach (online and in-person)
P04	• Use of remote communication Increased caution going out • Reduced social contact and limited stimulation • Stoic and resilient, keeping busy	• Use of remote communication has ‘become the norm’ • Continued caution going out; loss of personal freedoms due to general public’s disregard for safety • Stoic and resilient, keeping busy • Decline in communication skills; practicing in-person where possible
P06	• Use of internet (e.g. shopping) and remote communication • Dramatic change to diet and exercise to combat diabetes • Increased anxiety going out; avoiding others, practicing rigorous hygiene • Keeping busy at home	• Use of internet (e.g. shopping) and remote communication • Health changes not maintained to the same level • Continued anxiety to go out; less fixated on hygiene • Socially withdrawing; content at home Poor support from healthcare services; limited access
P07	• Limited impact as lives alone in rural location • Access to library limited but receiving books by delivery • Family visit infrequently; use of remote communication • Keeping busy, going out when rules allow, practising skills	• Significant decline in mental wellbeing (depression) • Increasingly isolated at home; not going out • Decline in dementia and physical wellbeing; doesn’t feel safe • Practising skills at home
P09	• Concerns over lost independence • Some in-person dementia groups have resumed • Keeping busy and active, taking each day as it comes • Helping others • Use of remote communication	• Uncertainty around Omicron variant and implications for future • Keeping busy and active • In-person dementia groups have resumed (blended approach) • Helping others, volunteering • Improved sense of community
	Post-Vaccine Rollout (Jan-Apr 2021)	(Dec 2021 – Jan 2022)
	P05	• Support from spouse and family (practical and emotional) • In-person dementia groups are closed • Routines aid focus	• Continued and increased support from spouse and family • In-person dementia groups have resumed; enjoys stimulation • Routines aid focus; increased use of memory aids • Increased episodes of confusion
P08	• Use of remote communication (social and dementia advocacy groups) • Isolated; lacking meaningful conversations • Decline in memory and thinking skills (e.g. word finding) • Exercise an important coping mechanism • Driving is ‘my freedom’	• Increasingly isolated; lacking meaningful conversations • Dementia advocacy and social groups permanently closed; lost contact with peers and advisers • Decline in communication skills • Exercise an important coping mechanism • Driving continues to facilitate independence

**Table 4.** Themes, subthemes and illustrative quotes about the ongoing impact of the coronavirus pandemic on people with dementia.

Themes	Subthemes	Illustrative quotes
1. Navigating a changing world: Living with coronavirus	1.1 restricted: ‘What I’m living now is no kind of life’	“you’ve got a choice and sort of say, ‘Oh, well, it would be nice to go to such-and-such a place’, and you think, ‘well, yeah, it’s nice but it’s too scary to do it’, so you don’t do it because it’s not a necessity. And so that’s wrong. That’s wrong. You shouldn’t be restricted from doing things through fear!” (P02)
	1.2 expanding: ‘it feels a bit more normal’	“You see people are much more aware than they were in previous times of the necessity to be immunised…And that was quite astounding how quickly that changed. And I thought it was very positive.” (P04) “Certainly, the groups that I’ve been involved with are slowly got back to normality, albeit reduced numbers.” (P09)
2. A ‘downward spiral’: Managing advancing dementia	2.1 deteriorating skills	“my verbal skills are not anymore as good as they used to be…it takes me more time to express something. So, I’m coming nearer the stage where you get locked in and you understand everything that is said, but to express it and react to it is more difficult. Because you lose your practise…Your skills go away if you don’t practise them” (P04)
	2.2 ‘Use it or lose it’: Strategies to maintain skills and independence	“Oh, yes, I’ve tried to keep my mind occupied, what’s left of it… It’s the only thing that there is, you know, to keep my mind at least ticking over. I don’t know what would happen if I didn’t do that.” (P07) “The more you learn…re-learn something, the more the brain has to work at it and get that. And the more you…the brain has to work, that keeps the dementia at bay, because your…you’re not letting it… it’s like I’m not going to let the dementia win” (P03)
3. Availability, accessibility and suitability of support	3.1 Support from family and friends	“A number of people – four or five people – will ring me at least once a week. And that’s a Godsend. Because I can speak with them and give them the update on how my wife is – not that much development usually – but it is nice to speak with somebody, as you can imagine.” (P01)
	3.2 Support from formal services	“The only thing which is a definite lack of support is from the GPs. There is no support whatsoever…I don’t even like ringing up because you feel you’re intruding” (P06) “for somebody with Alzheimer’s disease, I mean, you’ve still got your own personality. They’re not all…people aren’t all the same…I mean, they do have people who go round and talk to people, don’t they, and check in on them a couple of times a week? That’s nice for some people, but…it would drive me mad.” (P07)

### Navigating a Changing World: Living with Coronavirus

With public health restrictions and surging cases of the highly infectious Omicron variant in Britain, people with dementia were again being forced to adapt to a “whole new world” (P02) in December 2021 and January 2022. Reflecting on earlier experiences, attitudes, and coping strategies, this theme highlights the changes people with dementia had made almost 2 years into the pandemic and how some facets of their lives were being restricted, while others were expanding.

#### Restricted: ‘What I’m Living Now is no Kind of Life’

Despite increasing freedoms following the lifting of the most severe social restrictions, such as ‘stay at home’ orders (see Figure 1), coronavirus continued to limit participants’ lives. Uncertainty about the new Omicron variant dominated interviews conducted in December 2021, with concerns raised over national preparedness to cope, and what it could mean for public health and future plans:

“It's actually worse, in a way, because there's more unknown than when we spoke before...right now, today, we're in the unknown beginning of almost a whole new world...it feels even more uncomfortable and scary now.” (P02)

As time elapsed and Omicron became regarded as less “virulent in its effects than the other outbreaks” (P04), new concerns began to appear, with the public seen as being “much more laissez-faire about the way [they] behave in public than they used to be three or 4 months ago” (P04). Although mask-wearing and social distancing were no longer compulsory, disregard for these measures left several participants fearful of situations where they might encounter large groups of people, such as on public transport or at the theatre: “I don’t want to take those kind of chances” (P02).

For many, the transfer of responsibility from national, Government-imposed restrictions to the individual meant consciously limiting their meaningful activities, such as attending social events, exercise classes, travelling, and isolating themselves from society to protect themselves and others from potential harm.

“I have to be severely careful of how I go about it. That takes away a certain freedom which I would have had in the past...I have to think where I go, what time I go. What the places would be like.” (P04)

Indeed, seven participants were imposing additional restrictions over and above those required by law, such as not going out and avoiding crowds, and relying instead on solitary activities within the home such as knitting, reading, and watching television to remain occupied: “I sort of entertain myself a hell of a lot, really” (P08). After almost 2 years of restrictions, the impact of sustained isolation and limited stimulation had, perhaps unsurprisingly, led to a marked decline in mental wellbeing for some, most notably in those aged 81-90 who lived alone: “I don’t have any energy or enthusiasm to pick up a hobby of any sort” (P01).

“It’s getting a bit depressing...what I’m living now is no kind of life. I don’t enjoy my life at all and I’ve always enjoyed my life” (P07)

#### Expanding: ‘It feels a bit more normal’

While many faced challenges, there was also a sense that life was “getting better” (P08) as opportunities to socialise in-person and resume valued activities increased. Several dementia support programs had reconvened, offering stimulation and variety that was previously missing: “we’re back at the farm, and dancing…it’s nice to share it with other people” (P05). For some, adapting to these changing circumstances was a vital step towards achieving some semblance of normality: “you’ve got to get on with life…life is for living” (P05).

Recognising the importance of re-entering a previously restricted world, several participants living with young-onset dementia continued to rely on now habitual protective health measures to mitigate the risk of coronavirus. Masks, once considered to be an inconvenience, had become an “extra bandage” (P03) for personal safety, enabling this participant with anxiety to “get me out of the house” (P03). Furthermore, vaccinations, which bore little significance to participants in earlier interviews as restrictions kept them at home, were now viewed as a “necessity” (P04), and the national surge in uptake “for the benefit of the community” was “a beautiful thing to see” (P02). For several participants, this symbolised a positive shift in society and the reinstatement of a, feared lost, community spirit:

“There was a feel before COVID that our society was breaking down…and everybody was on the take rather than on the give; and suddenly…the spotlight has completely turned round” (P02)

During periods of stricter public health measures where social mixing was not permitted, remote technologies such as Zoom, FaceTime and WhatsApp were fundamental in maintaining participants’ relationships with family members and friends. Interestingly, while the return of in-person meetings was highly valued, seven participants had embraced a more blended approach to communication, as “modern technology” became “part of a norm” (P04). For those increasingly isolated at home, these virtual methods of communication could keep them connected in an otherwise shrinking world. Furthermore, the initial shift to online shopping during periods of strict national confinement became a permanent change for some, welcoming the greater convenience and choice it offered. Many participants also continued to spend time in nature, for example relaxing in private gardens and exercising in local parks, as it conferred additional benefits: “it keeps the mood up” (P08).

### A ‘Downward Spiral’: Managing Advancing Dementia

Initial fears of accelerated decline evident in earlier interviews were being put to the test as increasing opportunities for social interaction and meaningful activities arose. This theme therefore reflects participants’ new concerns and the strategies they were implementing to preserve skills and maintain their independence.

#### Deteriorating Skills

Almost 2 years into a world with social restrictions intended to protect physical health, participants’ earlier fears of accelerated decline and “suspicions of change have been confirmed” (P02). Reported losses were broad, including worsening memory, concentration, motivation, navigation, and administrative skills. This was a source of grave concern and frustration: “I have moments when I forget what I’m doing, or why I’m doing it. And I find that a bit frightening” (P05). For one participant who was socially withdrawing due to age, these changes meant meaningful, home-based activities such as woodwork became more difficult: “I do have to be careful what I’m doing now, because my brain no longer works in the same way that it used to” (P06).

The most notable losses were in communication skills as opportunities for social integration returned, highlighting the scale of the change to both themselves and others. For some, this decline has left them feeling “quite awkward with people,” reaching a point where “I can’t talk anymore. I can’t take it in anymore” (P08). Others were cognizant that they “now have a bigger disability” which, left unaddressed, could lead to further loss of skills, confidence, and isolation:

“…you talk less, because you don't want to be highlighting it. And that, then, is like a vicious downward spiral, isn't it? Because you're practising it less, you become even worse at it. And therefore, you avoid people more and more, and the level of isolation gets bigger and bigger” (P02)

This metaphorical ‘downward spiral’ was being realised for one of the oldest participants, for whom living alone was drastically becoming less viable as her dementia progressed and coronavirus restricted her already limited social interactions:

“It’s getting so much worse… of not knowing anything, not remembering anything, not being able to do anything… The virus is just an excuse to just sit here and rot…[and] the longer I don’t go out, the less I’m inclined to” (P07)

To address this observed decline and worsening emotional wellbeing, one participant thought support from someone professionally trained for himself and other people with dementia would be useful: “Even though they [people with dementia] still need it just as much as anybody else…it doesn’t exist” (P02).

#### ‘Use it or Lose it’: Strategies to Maintain Skills and Independence

Considering their declining abilities, there was a clear resolve among many participants to identify strategies to limit further decline: “I’m not going to let the dementia win” (P03). Typically, these were cognitive and functional strategies such as using memory aids, frequenting familiar places and practising affected skills, though some also focused on improving their physical health through diet and exercise (P06, P09). While similar strategies were discussed in earlier interviews, motivated by a ‘fear of decline’, the tone had shifted at this time-point, with greater significance placed on addressing the new realities of their dementia and re-evaluating what is possible as the world “opens up” (P02). For some, the renewed social freedoms were welcomed as they offered an opportunity to “combat” (P02) deteriorating communicative skills and practise meeting in-person, for example through peer support groups:

“The more you are with other people and talking with other people and listening to other people and fighting to be part of a conversation and that sort of thing, the more you're exercising those skills, the longer they will remain, despite the fact dementia is taking it away.” (P02)

Implementing these strategies was viewed as fundamental in continuing meaningful activities and therefore maintaining a sense of purpose. For example, several participants ascribed great meaning to driving, both to facilitate their independence in daily life, and as a measure of their cognitive abilities: “if I didn’t have the car, I wouldn’t go out at all” (P07) and “once I lose my driving licence, it means I’ve lost my mind, doesn’t it?” (P06). However, unlike earlier interviews, signs of dissonance were emerging in some, with some participants having to “override” (P06) their disinclination to practise skills and engage in stimulating activities: “it’s really easy to say ‘I don’t want to, but I have to, it’s been a hard day, but I have to,’ so…I usually just make myself do these things” (P03).

Some discussed strategies they used to compensate for their advancing dementia and had self-awareness of how their dispositions and past experiences influenced their ability to cope: “I’m very fortunate. I still have a lot of…educational capital that has helped me to do so well for such a long time. I’m pleased that I have that.” (P04).

### Availability, Accessibility and Suitability of Support

With new difficulties emerging in the context of an evolving pandemic and advancing dementia, participants discussed their support provisions at both a formal (i.e. health and social care) and informal (i.e. family, friends, neighbours) level. This theme therefore addresses the availability, accessibility, and suitability of participants’ support networks at this stage of the pandemic, including their recommendations for medium- and long-term improvements.

#### Informal Support

Finely-tuned coping strategies and pre-established informal networks meant several participants felt sufficiently supported and confident they had a “battery of people” (P01) they could call upon, and several spoke of how family, friends, and neighbours continued to offer invaluable social, emotional, and practical support. Inevitably, the degree and mode of support received varied by individual need, though the majority, including those most self-sufficient, reported continued social relationships via telephone, video calls, and, where comfortable, in-person meetings. Others appreciated more tangible support, such as domestic cleaning, shopping deliveries, and accompaniment to medical appointments: “luckily, she was allowed to come with me…otherwise…I’d be still walking around now looking for [the hospital department]” (P03).

For some participants facing additional barriers to their independence, such as immobility and reticence to go out, virtual contact from family and friends was considered a “Godsend” (P01) in keeping them connected with the outside world. For others, the easing of restrictions revealed gaps in their support networks, highlighting new needs: “that’s what I’d like…social interactions” (P08). Despite recognising these growing needs, older participants who lived alone were hesitant about asking for and accepting informal support. One participant who was staunchly independent and repeatedly affirmed “I don’t want anybody looking after me” (P07) had declined markedly since her interview 12 months prior. She commented “I’m not really fit to be on my own now” (see Table 3). She continued to express her wish to be independent, saying for example “I’m not going anywhere…to the bloody hospital or [having] a carer or anything like that.” This determination to avoid receiving support raised questions about how she would cope in the future.

#### Formal Support

Inequalities and variability in access to support were apparent in participants’ experiences of both health and social care. Perhaps unsurprisingly, five participants with existing connections to individuals and organisations, including charities, local authorities, dementia support programs, advocacy groups, designated healthcare professionals and welfare calls, were more likely to be receiving continued support or, at the very least, knew where they could go should they require it:

“luckily…because of all the business work I do in the dementia community, I actually have individual contact, or through a group” (P02).

Several of these participants attended various virtual and in-person groups, appreciating the social, emotional and practical support they offered. In stark contrast, one participant with young-onset dementia had endured permanent closures of valued support programs during the pandemic, leaving her with limited opportunities for stimulation and peer support from people who “get it” (P08). Reconfiguration of a national dementia charity, forced by financial losses during the pandemic, left this participant feeling abandoned, as phone numbers she had for advisers and group organisers no longer worked making it “quite difficult to get the information…you’ve got to go looking for it…But it’s got to be life or death for me to make a phone call” (P08). Asked whether a more proactive approach from charities would be an effective form of support, she agreed:

“Unless I write a note to myself and then go to the computer and email them, it isn’t going to happen. Whereas…if I get an email come through with a bit of information on it, then that…primes the memory” (P08)

Notably, several participants were not receiving any social support for their dementia at this stage of the pandemic, instead demonstrating signs of social withdrawal: “I can’t do with socialising. No, people get on my nerves” (P07).

Since initial accounts were collected early in the pandemic, difficulties with healthcare services had dominated participants’ experiences (see Table 3). Many of these difficulties, including lengthy delays in medical appointments and difficulties seeing usual medical staff were still evident almost 2 years on, causing some to have “absolutely no faith” (P01) in their primary care provision:

“If you’re sort of at death’s door where you can have a [telephone] appointment in three weeks but we’re still not going to see you, it doesn’t instil confidence, does it?” (P06)

Despite this, the majority of participants had in fact accessed some form of healthcare (e.g. dentists, general practitioners, hospital appointments) in recent months, suggesting that availability had increased. Many valued continuity of care with known health professionals as their familiarity with dementia instilled confidence about accurate assessments, most commonly for driving licence renewals, and management of health conditions. Some even willingly accepted delays so that they could see their ‘own’ practitioner:

“the practitioner knows me, and I know him. And that makes it much easier to diagnose things more quickly” (P04)

However, these positives were often negated by the perceived unsuitability of remote consultations and telephone triage by individuals that were not “dementia trained” (P02). For example, participants continued to feel they were unable to adequately express themselves by telephone due to forgetting key information, were rushed by time-limited appointments, and felt unheard by practitioners:

“Just take their time and wait a minute, basically. Because sometimes, if they rush you…I just find it more confusing” (P05)

Participants’ recommendations for improving healthcare experiences therefore sought to address these difficulties and re-establish a prompt and person-centred approach.

## Discussion and Implications

To our knowledge, this is the first qualitative study to compare the experiences of people with dementia across different stages of the coronavirus pandemic. Our findings centred on narratives of restriction and expansion as attempts to re-enter society were met with overwhelming caution and the compromising of valued activities. Periods of protracted isolation and loss of meaningful pastimes were believed to contribute to worsening of cognitive, functional, and psychological wellbeing; however, there was clear resolve to identify and implement strategies to mitigate these changes. Furthermore, existing difficulties such as disparate access to support, and limited social networks had been amplified by the pandemic, raising questions about the preparedness of people with dementia to re-enter this new world without adequate support. Our findings have implications for future restrictions or new pandemics, and wider implications for support needs for people with dementia which the pandemic has served to highlight further.

In keeping with previous research early in the pandemic (Suarez-Gonzalez et al., 2021), perceptions of accelerated decline in cognitive and functional domains were apparent, although changes in cognition and function during the pandemic were to be expected, given the progressive nature of dementia (Clare et al., 2022). Initial fears that skills had been lost were, in part, realised by participants in this study; they reported that increased opportunities for social reintegration and resumption of some meaningful activities revealed marked changes in communication and language abilities. Difficulties in remaining mentally stimulated and avoiding a vicious ‘downward spiral’ of increasing disability, mood, and further isolation were compounded by self-imposed cautious behaviour associated with fears of catching the Omicron variant, even when statutory restrictions were lifted. Our findings uphold early concerns about the impact social isolation may have on psychosocial wellbeing (Clare et al., 2022; Pentecost et al., 2022; Rising et al., 2022), highlighting the distress caused by awareness of negative changes attributed to reduced social activity and doubts about whether such skills can be restored. Encouragingly, this study, similar to earlier research (O'Rourke et al., 2021; Pentecost et al., 2022; Stapley et al., 2022), found people with dementia continued to exhibit signs of resilience, responding to new challenges by identifying new and existing strategies to support everyday life with dementia.

Nonetheless, it is vital that, in the event of a future pandemic, people with dementia are proactively offered advice on coping strategies to empower them to adjust to protracted periods of social restrictions and any associated anxiety in order to increase the likelihood of successful adaptation and the continuation of meaningful activities that support their ability to live well. As we emerge from the pandemic and to counter the ‘downward spiral’ of the effects of self-imposed cautious re-engagement with the world, welfare checks could, for example, offer support for emotional wellbeing, strategies for contingency planning, and advice on social engagement (Tuijt et al., 2021; Yates et al., 2019). A recent meta-synthesis found that, provided they have access to sufficient resources, people with dementia can effectively maintain continuity in their lives (Górska et al., 2018).

However, our findings also highlight the longer-term disruption of the pandemic on resource provision, emphasising how important these are to people living with dementia. Initial closures of valued in-person dementia support groups early in the pandemic were a source of distress and anxiety for many people with dementia (Pentecost et al., 2022). Twenty-two months on, this study found that only some of these groups have resumed while others have permanently closed. Dementia support programs can provide people with meaningful activities and a sense of purpose, offer a source of valuable peer support, and grant access to volunteers and trained advisers, all of which can improve self-confidence and independence (Clarke et al., 2013; Weetch et al., 2021). For those groups that have restarted, a ‘blended’ online and in-person approach has been widely adopted; generally, this has been well-received for those who can gain access (Masoud et al., 2021). We also found that people with dementia with pre-existing connections to formal services and peer support groups were less adversely affected than those with limited social networks, perhaps due to perceived or actual availability of support (Pentecost et al., 2022; Smaling et al., 2022), indicating another disparity in access to support which needs to be addressed. Although pre-dating the pandemic, such inequities have been magnified by it. Focusing on integrated systems of post-diagnostic support in primary care (Bamford et al., 2021) may help to ensure people with dementia and their families are not missed at any time, and especially during times of public health crisis.

By exploring differences in the experiences of people with dementia over time, this study offers new insights into the ongoing impact of social restrictions during the coronavirus pandemic and its possible legacies. Nevertheless, it is not without limitations. In order to achieve our comparison, recruitment was limited to a pool of participants who had previously taken part in IDEAL-CDI or INCLUDE interviews (O'Rourke et al., 2021; Pentecost et al., 2022; Stapley et al., 2022) and were able to be interviewed at this time point. The sociodemographic profile of our sample was limited to white British individuals mostly living with young-onset dementia, and four of the nine participants were University educated. Capturing the experiences of people living with young-onset dementia is a strength of this study; however, it is important to acknowledge that those individuals with more severe dementia or from other ethnic groups or backgrounds may have faced unique challenges not accounted for by our findings (O'Rourke et al., 2021). Notwithstanding, our findings offer novel insights into the effects of changing social restrictions on people with dementia 22 months into the pandemic in the UK, highlighting the complexities of navigating a new world with variable support and cognitive resources.

## Conclusions

Although people with dementia demonstrated resilience and a capacity to adapt to the pandemic, mitigation of the negative consequences of restrictions highlighted in this study will likely necessitate some form of coordinated effort from people with dementia, their family members and friends, and adequately funded health and social services moving forward. However, as this and earlier studies demonstrate, existing inequalities in service availability and suitability, including delays in receiving care, logistical barriers, diminished confidence in providers, and closures of dementia support programs, will need to be addressed in order to offer equal and effective support to people living with dementia (Tuijt et al., 2021; Van Horik et al., 2022). Certainly, without timely, proactive, and personalised care, social isolation and disablement is likely to continue unchecked, thereby limiting the ability of people with dementia to reintegrate into society as they choose. Therefore, as communities look to rebuild these support networks and services, it is critical we learn from the experience of the pandemic and do not miss the opportunity at an individual, societal, and political level to improve the availability, accessibility and suitability of advice, support, and social interaction which will ensure people with dementia are sufficiently supported and empowered to live well.

## Supplemental Material

Supplemental Material - Navigating the coronavirus pandemic 2 years on: Experiences of people with dementia from the British IDEAL cohort Click here for additional data file. Supplemental Material for Navigating the coronavirus pandemic 2 years on: Experiences of people with dementia from the British IDEAL cohort by Eleanor Dawson, Rachel Collins, Claire Pentecost and Sally Stapley, Catherine Quinn, Catherine Charlwood, Christina Victor and Linda Clare in Dementia

## References

[bibr1-14713012231158215] Alzheimer’s Society . (2022, March 28). Person-centred care. https://www.alzheimers.org.uk/about-dementia/treatments/person-centred-care

[bibr2-14713012231158215] Bamford C. Wheatley A. Brunskill G. Booi L. Allan L. Banerjee S Harrison Dening K. Manthorpe J. Robinson L. PriDem study team on behalf of the PriDem Study Team (2021). Harrison DeningK.ManthorpeJ.RobinsonL.Key components of post-diagnostic support for people with dementia and their carers: A qualitative study. Plos One, 16(12), Articlee0260506. 10.1371/journal.pone.0260506 34928972 PMC8687564

[bibr3-14713012231158215] Banerjee D. Rai M. (2020). Social isolation in Covid-19: The impact of loneliness. The International journal of social psychiatry, 66(6), 525–527. 10.1177/0020764020922269 32349580 PMC7405628

[bibr4-14713012231158215] Bannon S. M. Wang K. E. Grunberg V. A. Dickerson B. C. Vranceanu A.-M. (2022). Couples’ experiences managing young-onset dementia early in the Covid-19 pandemic. The Gerontologist, 62(8), 1173–1184. 10.1093/geront/gnab162 34739072 PMC9451019

[bibr5-14713012231158215] Borelli W. V. Augustin M. C. de Oliveira P. B. F. Reggiani L. C. Bandeira-de-Mello R. G. Schumacher-Schuh A. F. Chaves M. L. F. Castilhos R. M. (2021). Neuropsychiatric symptoms in patients with dementia associated with increased psychological distress in caregivers during the Covid-19 pandemic. Journal of Alzheimer's disease: JAD, 80(4), 1705–1712. 10.3233/jad-201513 33646168

[bibr6-14713012231158215] Boutoleau-Bretonnière C. Pouclet-Courtemanche H. Gillet A. Bernard A. Deruet A. L. Gouraud I. Mazoue A. Lamy E. Rocher L. Kapogiannis D. El Haj M. (2020). The effects of confinement on neuropsychiatric symptoms in alzheimer's disease during the Covid-19 crisis. Journal of Alzheimer's disease: JAD, 76(1), 41–47. 10.3233/jad-200604 32568211 PMC9988367

[bibr7-14713012231158215] Brown E. E. Kumar S. Rajji T. K. Pollock B. G. Mulsant B. H. (2020). Anticipating and mitigating the impact of the Covid-19 pandemic on alzheimer's disease and related dementias. The American Journal of Geriatric Psychiatry: Official Journal of the American Association for Geriatric Psychiatry, 28(7), 712–721. 10.1016/j.jagp.2020.04.010 32331845 PMC7165101

[bibr8-14713012231158215] Canevelli M. Valletta M. Toccaceli Blasi M. Remoli G. Sarti G. Nuti F. Sciancalepore F. Ruberti E. Cesari M. Bruno G. (2020). Facing dementia during the Covid-19 outbreak. Journal of the American Geriatrics Society, 68(8), 1673–1676. 10.1111/jgs.16644 32516441 PMC7300919

[bibr9-14713012231158215] Clare L. Martyr A. Gamble L. D. Pentecost C. Collins R. Dawson E. Hunt A. Parker S. Allan L. Burns A. Hillman A. Litherland R. Quinn C. Matthews F. E. Victor C. (2022). Impact of COVID-19 on 'living well' with mild-to-moderate dementia in the community: Findings from the IDEAL cohort. Journal of Alzheimer's disease: JAD, 85(2), 925–940. 10.3233/jad-215095 34776448

[bibr10-14713012231158215] Clare L. Nelis S. M. Quinn C. Martyr A. Henderson C. Hindle J. V. Jones I. R. Jones R. W. Knapp M. Kopelman M. D. Morris R. G. Pickett J. A. Rusted J. M. Savitch N. M. Thom J. M. Victor C. R. (2014). Improving the experience of dementia and enhancing active life–living well with dementia: Study protocol for the IDEAL study. Health and quality of life outcomes, 12(164), 164. 10.1186/s12955-014-0164-6 25433373 PMC4260182

[bibr12-14713012231158215] Clarke C. Keyes S. Wilkinson H. Alexjuk J. Wilcockson J. Robinson L. Reynolds J. McClelland S. Hodgson P. Corner L. Cattan M. (2013, October 9). Healthbridge: The national evaluation of peer support networks and dementia advisers in implementation of the national dementia strategy for England. https://www.gov.uk/government/publications/peer-support-networks-and-dementia-advisers-evaluation

[bibr13-14713012231158215] Cousins E. de Vries K. Harrison Dening K. (2022). ‘Four walls and a garden': Exploring the experiences of families affected by dementia during the COVID-19 pandemic. Dementia, 21(3), 810–829. DOI: 10.1177/14713012211059021 34918956 PMC8995930

[bibr14-14713012231158215] Dementia UK . (2022, June 30). Young onset dementia. https://www.dementiauk.org/about-dementia/young-onset-dementia/

[bibr15-14713012231158215] Di Lorito C. Masud T. Gladman J. Godfrey M. Dunlop M. Bosco A. Harwood R. H. (2021). Deconditioning in people living with dementia during the COVID-19 pandemic: Qualitative study from the promoting activity, independence and stability in early dementia (PrAISED) process evaluation. BMC geriatrics, 21(1), 529. 10.1186/s12877-021-02451-z 34620129 PMC8495442

[bibr16-14713012231158215] Gale N. K. Heath G. Cameron E. Rashid S. Redwood S. (2013). Using the framework method for the analysis of qualitative data in multi-disciplinary health research. BMC Medical Research Methodology, 13(1), 117. 10.1186/1471-2288-13-117 24047204 PMC3848812

[bibr17-14713012231158215] Giebel C. Cannon J. Hanna K. Butchard S. Eley R. Gaughan A. Komuravelli A. Shenton J. Callaghan S. Tetlow H. Limbert S. Whittington R. Rogers C. Rajagopal M. Ward K. Shaw L. Corcoran R. Bennett K. Gabbay M. (2021). Impact of COVID-19 related social support service closures on people with dementia and unpaid carers: A qualitative study. Aging & Mental Health, 25(7), 1281–1288. 10.1080/13607863.2020.1822292 32954794

[bibr18-14713012231158215] Górska S. Forsyth K. Maciver D. (2018). Living with dementia: A meta-synthesis of qualitative research on the lived experience. The Gerontologist, 58(3), e180–e196. 10.1093/geront/gnw195 28069886 PMC5946830

[bibr19-14713012231158215] Hanna K. Giebel C. Tetlow H. Ward K. Shenton J. Cannon J. Komuravelli A. Gaughan A. Eley R. Rogers C. Rajagopal M. Limbert S. Callaghan S. Whittington R. Butchard S. Shaw L. Gabbay M. (2022). Emotional and mental wellbeing following Covid-19 public health measures on people living with dementia and carers. Journal of Geriatric Psychiatry and Neurology, 35(3), 344–352. 10.1177/0891988721996816 33626977 PMC8996307

[bibr20-14713012231158215] Horik J. O. Collins R. Martyr A. Henderson C. Jones R. W. Knapp M. Quinn C. Thom J. M. Victor C. Clare L. IDEAL Programme Team (2022). Limited receipt of support services among people with mild-to-moderate dementia: Findings from the IDEAL cohort. International Journal of Geriatric Psychiatry, 37(3), 1–8. 10.1002/gps.5688 PMC9306706 35128725

[bibr21-14713012231158215] Masoud S. S. Meyer K. N. Martin Sweet L. Prado P. J. White C. L. (2021). “We don't feel so alone”: A qualitative study of virtual memory cafés to support social connectedness among individuals living with dementia and care partners during COVID-19. Frontiers in Public Health, 9: 660144. 10.3389/fpubh.2021.660144 34055724 PMC8155306

[bibr22-14713012231158215] Office for National Statistics . (2022, July 1). Coronavirus (COVID-19): Latest data and analysis on coronavirus (COVID-19) in the UK and its effect on the economy and society. https://www.ons.gov.uk/peoplepopulationandcommunity/healthandsocialcare/conditionsanddiseases

[bibr23-14713012231158215] O'Rourke G. Pentecost C. van den Heuvel E. Victor C. Quinn C. Hillman A. Litherland R. Clare L. (2021). Living with dementia under COVID-19 restrictions: Coping and support needs among people with dementia and carers from the IDEAL cohort. Ageing and Society, 1–23. 10.1017/S0144686X21001719

[bibr24-14713012231158215] Pentecost C. Collins R. Stapley S. Victor C. Quinn C. Hillman A. Litherland R. Allan L. Clare L. (2022). Effects of social restrictions on people with dementia and carers during the pre-vaccine phase of the COVID-19 pandemic: Experiences of IDEAL cohort participants. Health & social care in the community, 30(6), Article e4594–e4604. 10.1111/hsc.13863 35695217 PMC9349761

[bibr25-14713012231158215] Portacolone E. Chodos A. Halpern J. Covinsky K. E. Keiser S. Fung J. Rivera E. Tran T. Bykhovsky C. Johnson J. K. (2021). The effects of the Covid-19 pandemic on the lived experience of diverse older adults living alone with cognitive impairment. The Gerontologist, 61(2), 251–261. 10.1093/geront/gnaa201 33404634 PMC7901518

[bibr26-14713012231158215] Quinn C. Gamble L. D. Parker S. Martyr A. Collins R. Victor C. Dawson E. Hunt A. Pentecost C. Allan L. Clare L. (2022). Impact of COVID-19 on carers of people with dementia in the community: Findings from the British IDEAL cohort. International Journal of Geriatric Psychiatry, 37(5), 1–11. 10.1002/gps.5708 PMC9087398 35394090

[bibr27-14713012231158215] Rising K. L. Salcedo V. J. Amadio G. Casten R. Chang A. Gentsch A. O'Hayer C. V. Sarpoulaki N. Worster B. Gerolamo A. M. (2022). Living through the pandemic: The voices of persons with dementia and their caregivers. Journal of applied gerontology: the official journal of the Southern Gerontological Society, 41(1), 30–35. 10.1177/07334648211036399 34344205

[bibr28-14713012231158215] Sabatini S. Bennett H. Q. Martyr A. Collins R. Gamble L. D. Matthews F. E. Pentecost C. Dawson E. Hunt A. Parker S. Allan L. Burns A. Litherland R. Quinn C. Clare L. (2022). Minimal impact of COVID-19 pandemic on the mental health and wellbeing of people living with dementia: Analysis of matched longitudinal data from the IDEAL study. Frontiers in Psychiatry, 13: 849808. 10.3389/fpsyt.2022.849808 35370851 PMC8965515

[bibr30-14713012231158215] Silarova B. Nelis S. M. Ashworth R. M. Ballard C. Bieńkiewicz M. Henderson C. Hillman A. Hindle J. V. Hughes J. C. Lamont R. A. Litherland R. Jones I. R. Jones R. W. Knapp M. Kotting P. Martyr A. Matthews F. E. Morris R. G. Quinn C. Clare L. (2018). Protocol for the IDEAL-2 longitudinal study: Following the experiences of people with dementia and their primary carers to understand what contributes to living well with dementia and enhances active life. BMC public health, 18(1), 1214. 10.1186/s12889-018-6129-7 30376832 PMC6208177

[bibr31-14713012231158215] Smaling H. J. A. Tilburgs B. Achterberg W. P. Visser M. (2022). The impact of social distancing due to the Covid-19 pandemic on people with dementia, family carers and healthcare professionals: A qualitative study. International Journal of Environmental Research and Public Health, 19(1), 519. DOI: 10.3390/ijerph19010519 35010779 PMC8744737

[bibr32-14713012231158215] Stapley S. Pentecost C. Collins R. Quinn C. Dawson E. Morris R. G. Sabatini S. Thom J. Clare L. (2022). Living with dementia during the COVID-19 pandemic: Insights into identity from the IDEAL cohort. Aging and Society, 1–25. 10.1017/S0144686X22001350 10.1017/S0144686X22001350

[bibr33-14713012231158215] Suárez-González A. Rajagopalan J. Livingston G. Alladi S. (2021). The effect of COVID-19 isolation measures on the cognition and mental health of people living with dementia: A rapid systematic review of one year of quantitative evidence. EClinicalMedicine, 39(101047), 101047. 10.1016/j.eclinm.2021.101047 34386758 PMC8342894

[bibr34-14713012231158215] Talbot C. V. Briggs P. (2021). 'Getting back to normality seems as big of a step as going into lockdown': The impact of the COVID-19 pandemic on people with early to middle stage dementia. Age and Ageing, 50(3), 657–663. 10.1093/ageing/afab012 33481988 PMC7929391

[bibr35-14713012231158215] Tuijt R. Rait G. Frost R. Wilcock J. Manthorpe J. Walters K. (2021). Remote primary care consultations for people living with dementia during the COVID-19 pandemic: Experiences of people living with dementia and their carers. The British journal of general practice: the journal of the Royal College of General Practitioners, 71(709), Article e574–e582. 10.3399/BJGP.2020.1094 33630749 PMC8136581

[bibr36-14713012231158215] Weetch J. O’Dwyer S. Clare L. (2021). The involvement of people with dementia in advocacy: A systematic narrative review. Aging & Mental Health, 25(9), 1595–1604. 10.1080/13607863.2020.1783512 32578451

[bibr37-14713012231158215] World Health Organisation . (2020, March 11). WHO Director-General's opening remarks at the media briefing on COVID-19-11 March 2020. WHO. https://www.who.int/director-general/speeches/detail/who-director-general-s-opening-remarks-at-the-media-briefing-on-covid-19–11-march-2020

[bibr38-14713012231158215] World Health Organisation . (2021, September 2). Dementia: Key facts. WHO. https://www.who.int/news-room/fact-sheets/detail/dementia

[bibr39-14713012231158215] Yates L. Csipke E. Moniz-Cook E. Leung P. Walton H. Charlesworth G. Spector A. Hogervorst E. Mountain G. Orrell M. (2019). The development of the Promoting Independence in Dementia (PRIDE) intervention to enhance independence in dementia. Clinical Interventions in Aging, 14(14), 1615–1630. 10.2147/CIA.S214367 31571842 PMC6748161

