# Supplementary material for: Navigating the coronavirus pandemic 2 years on: Experiences of people with dementia from the British IDEAL cohort
Source: Dementia (London). 2023 Feb 24;22(4):760–82. doi: 10.1177/14713012231158215 (PMC9969185; doi:10.1177/14713012231158215)
Supplement: Supplemental Material - Navigating the coronavirus pandemic 2 years on: Experiences of people with dementia from the British IDEAL cohort [file sj-pdf-1-dem-10.1177_14713012231158215.pdf]

Topic Guide

Topic	Main Questions	Prompts/Probes (not exhaustive)
Difficulties or changes to daily routines	Is life getting 'back to normal' for you yet? (How/why not?)<br><br>Are you able to do everything you want to do at the moment?	Ability to go out<br>Seeing friends and family<br>Omicron variant and Coronavirus restrictions<br>What would help life get 'back to normal'?<br>Specific prompts/probes (e.g. 'When you spoke to [researcher] last time, you were hoping to return to your swimming club…')<br>Hobbies, activities, dementia support groups<br>Specific prompts (e.g. fear of decline; loss of self-confidence; returning to normal; reconnecting with the world)
Own coping strategies and/or support found to be helpful	How do you feel you are coping now, at this stage of the pandemic?<br><br>What's the easiest thing to cope with now?<br><br>What's the hardest thing to cope with now?	Specific prompts (e.g. living alone, young-onset dementia)<br><br>**> If coping: what helps you to cope?** (e.g. strong/resilient person; learning something new; Zoom calls with family)<br>Specific prompts if last interview was positive<br><br>**> If not coping: why is this? What would help you to cope?** (e.g. help from family, friends, neighbours, local community, GP, memory service, charities)<br>Specific prompts if last interview was negative<br><br>Specific prompts (e.g. less depressed or anxious; more time with their partner; quieter; less pressure to go out; spent a lot of time at home before the pandemic)<br><br>Specific prompts (e.g. loneliness; social isolation)
Additional or missing support or information that would be helpful	How is your access to healthcare services, such as GP appointments, now?<br><br>What information or support do you need now that you aren't getting?	Suitability, availability, accessibility<br>Continuity of care<br>Specific prompts (e.g. communication and choice, appointment modalities)<br><br>Practical and/or emotional support<br>'Checking in' calls<br>Specific prompts (e.g. absence of support; dementia identity and self-advocacy; 'pro-active' service support; personalised support; practical support)
Any unexpected benefits or outcomes; and how they might be maintained	Has there been anything positive to come out of the pandemic?	More activities with partner<br>Specific prompts (e.g. connected communities, support from or getting to know neighbours)
Training or information health or social care professionals or volunteers need to help people with memory difficulties in the COVID-19 situation	If you could give a healthcare professional (e.g. a GP) or someone like a volunteer one piece of advice to best help people with memory problems at this point of the pandemic, what would it be?	Specific prompts (e.g. dementia identity and self-advocacy; 'pro-active' service support; personalised dementia service support; practical support; training)
